# Effect of Plant-Derived n-3 Polyunsaturated Fatty Acids on Blood Lipids and Gut Microbiota: A Double-Blind Randomized Controlled Trial

**DOI:** 10.3389/fnut.2022.830960

**Published:** 2022-02-11

**Authors:** Hongjie Liu, Xiaoqin Li, Yalun Zhu, Yue Huang, Qin Zhang, Shan Lin, Can Fang, Linyan Li, Yanling Lv, Wenhua Mei, Xiaolin Peng, Jiawei Yin, Liegang Liu

**Affiliations:** ^1^Department of Nutrition and Food Hygiene, Hubei Key Laboratory of Food Nutrition and Safety, School of Public Health, Tongji Medical College, Huazhong University of Science and Technology, Wuhan, China; ^2^MOE Key Lab of Environment and Health, School of Public Health, Tongji Medical College, Huazhong University of Science and Technology, Wuhan, China; ^3^Zhuhai Center for Disease Control and Prevention, Zhuhai, China; ^4^Shenzhen Nanshan Centre for Chronic Disease Control, Shenzhen, China

**Keywords:** n-3 polyunsaturated fatty acids, hyperlipidemia, gut microbiota, randomized controlled trial, blood lipid

## Abstract

**Background:**

Several cardioprotective mechanisms attributed to n-3 polyunsaturated fatty acids (PUFAs) have been widely documented. Significant interest has recently focused on the role of human gut microbiota in metabolic disorders. However, the role of plant-derived n-3 PUFAs on blood lipid profiles is controversial and the effect on gut microbiota is still unclear.

**Objectives:**

We aimed to perform a double-blind randomized controlled trial to test the effect of plant-derived n-3 PUFAs on the blood lipids and gut microbiota of patients with marginal hyperlipidemia.

**Methods:**

According to the inclusion and exclusion criteria, 75 participants with marginal hyperlipidemia were randomly assigned to the intervention group (supplied with n-3 PUFA-enriched plant oil) or control group (supplied with corn oil), respectively, for a 3-month treatment. Participants and assessors were blinded to the allocation. The primary outcomes of the trial were the changes in serum lipid levels. Secondary outcomes were changes in gut microbiota and metabolites. For the primary outcomes, we conducted both an intent-to-treat (ITT) analysis and a per protocol (PP) analysis. For the secondary outcomes, we only conducted the PP analysis among the participants who provided fecal sample.

**Results:**

Fifty-one participants completed the trial. Relative to the control group, the n-3 PUFA supplementation resulted in significant reduction in total cholesterol (TC) levels (−0.43 mmol/L, 95% CI−0.84 to−0.01 mmol/L, *P* < 0.05). The n-3 PUFA supplementation was also associated with significantly increased relative abundance of *Bacteroidetes* in phylum level (*P* < 0.01; false discovery rate (FDR) corrected *p* = 0.11), and decreased the ratio between *Firmicutes* and *Bacteroidetes* (*P* < 0.05; FDR corrected *p* = 0.16). At genus level, the intervention of plant derived n-3 PUFAs resulted in a significant decrease in relative abundance of *Phascolarctobacterium* (*P* < 0.01; FDR corrected *p* = 0.18) and *Veillonella* (*P* < 0.01; FDR corrected *p* = 0.18) after the intervention.

**Conclusions:**

Our results demonstrated that plant-derived n-3 PUFAs beneficially affected the serum levels of TC and decreased the ratio between *Firmicutes* and *Bacteroidetes* during the 12-week intervention period, which might confer advantageous consequences for lipid metabolism and intestinal health.

## Introduction

Hyperlipidemia is an important risk factor for coronary heart disease and stroke (1). According to the data released by the China National Stroke Screening and Prevention Project, the standardized prevalence of hyperlipidemia was 43% among Chinese adults of 40 years or above (2). Adults with prolonged exposure to even moderate hyperlipidemia may benefit from more aggressive primary prevention (3). Epidemiological studies indicated that sensible management of diet played an important role to relieve the condition of hyperlipidemia (4).

Habitual fat intake is closely related to lipid metabolism and cardiometabolic health (5). Considerable research supports a reduction in cardiovascular disease risk with habitual dietary intake and high blood circulation of n-3 polyunsaturated fatty acids (PUFAs) (6, 7). The primary source of eicosapentaenoic acid (EPA), docosapentaenoic acid (DPA) and docosahexaenoic acid (DHA) is seafood especially oily fish. However, realizing the goal of fatty fish consumption is obstructed by the food preferences and by concerns about the bio-enrichment of heavy metals (8). This has increased the interest in the health benefits of n-3 PUFAs derived from plants, including alpha-linolenic acid (ALA) from flaxseed, rapeseed, and camelina oils, and stearidonic acid (SDA) from echium oil (9). The role of plant-derived n-3 PUFAs in the of lipid metabolism has not been well illustrated and their effects on blood lipid profiles are still controversial (10–15).

In recent years, significant interest has focused on the presence of characteristic gut microbiota, specific gut microbiota-dependent pathways, and downstream metabolites in cardiovascular disease and metabolic disorders (16, 17). Previous cross-sectional study has identified 34 gut bacterial taxa associated with blood lipid levels among healthy subjects (18). Another case-control study demonstrated that hypercholesterolemia patients showed a particular gut bacterial signature, which characterized by lower relative abundance of both *Anaeroplasma* and *Haemophilus* and higher relative abundance of both *Odoribacter* and *Ruminococcus* compared with the control group (19). In addition, short-chain fatty acids (SCFAs) such as isobutyric acid correlated positively with *Odoribacter* and lipid parameters (19). These prior studies suggested the important role of gut microbiota in the variation of blood lipid levels, and supported the potential of therapies altering the gut microbiome to improve hyperlipidemia status. A previous review summarized a potential positive action of marine-derived n-3 PUFAs on gut microbiota (20). However, limited studies have evaluated how the plant-derived n-3 PUFAs related to the gut microbiota, and existing results were inconsistent (21–23).

Therefore, we aimed to perform a double-blind randomized controlled trial to test the effect of plant-derived n-3 PUFAs on the blood lipids and gut microbiota of patients with marginal hyperlipidemia. The primary outcomes of the trial were the changes in serum lipid levels. Secondary outcomes were changes in gut microbiota and metabolites.

## Subjects and Methods

### Study Population

The institutional research ethics board at Tongji Medical College approved the study protocol. All participants provided written informed consent prior to enrollment.

**Figure 1:**
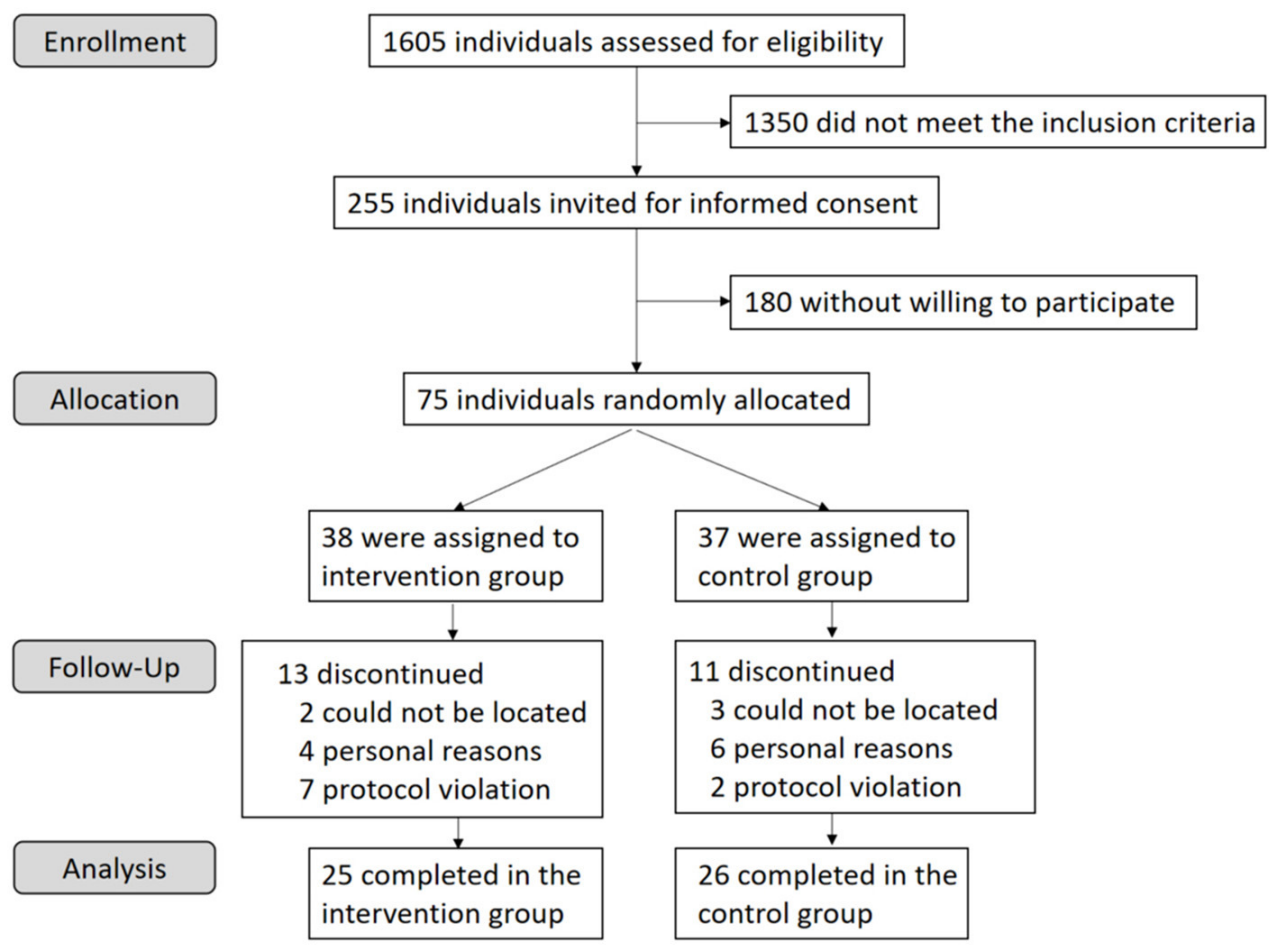
Flow chart of the participant recruitment and withdraw.

The study recruited 1,605 individuals aged 30–65 years from a community health service center in Nanshan district, Shenzhen, China from July 2018 to September 2018. We collected the data through questionnaire, physical examination, laboratory detection, and clinical records. Participants were required to be native Chinese who lived in Shenzhen. After a screening blood lipid test, subjects with marginal hyperlipidemia (total cholesterol (TC) ≥ 5.2 mmol/L or triglycerides (TG) ≥ 1.7 mmol/L) were considered to meet the preliminary inclusion criteria according to the Chinese Guidelines on Prevention and Treatment of Dyslipidemia (24). The exclusion criteria included used flaxseed/rapseed/soybean oil as daily cooking oil, consumed walnuts or fatty fish more than 100 g/week, drank alcohol more than 2 drinks/day, smoked more than 10 cigarettes/day, had food allergies, had a history of chronic or metabolic diseases, used medication or supplements known to affect blood lipids, took antibiotics for more than three consecutive days in the last month. Women who were not menopausal or went into menopause for <1 year were excluded. A total of 225 individuals meeting the inclusion and exclusion criteria were contacted to participate in the study. Finally, 75 individuals were willing to participate in the study (Figure 1). The study was registered at clinicaltrials.gov as HPG2017102074.

### Sample Size Calculation

In a repeated measures design, sample size estimation was based on detecting a treatment difference in TC change of 0.60 with an SD of 0.80. With type I error (α) of 0.05 and type II error (β) of 0.20 (power = 80%), using *r* = 0.8 to account for the high degree of correlation between successive measures, a required sample size of 25 patients per group was obtained, which increased to 35 patients for potential dropouts (30%). However, because of a larger-than-expected dropout at the start, participant recruitment numbers were increased to 75.

### Study Design

In this double-blind randomized controlled trial, after stratification by sex and TC <6.2 mmol/L or ≥ 6.2 mmol/L (240 mg/dL) but without a predetermined block size, participants were randomly and equally assigned to two groups in a blinded fashion. According to the randomization scheme, subjects were allocated to the intervention or control group, and started supplementation with n-3 PUFA oil capsule or corn oil capsule, respectively. During the intervention period of 12 weeks, subjects in the intervention group consumed daily 4 g of n-3 PUFA-rich oil, of which 9.6% from saturated fatty acids, 15.7% from monounsaturated fatty acids, 24.9% from n-6 polyunsaturated fatty acids, and 41.2% from n-3 PUFAs (1.2 g ALA and 0.4 g SDA). Subjects in the control group consumed daily 4 g of corn oil, of which 13.1% from saturated fatty acids, 28.9% from monounsaturated fatty acids, 44.4% from n-6 polyunsaturated fatty acids, and 0.6% from n-3 PUFAs (n-3 PUFAs < 0.05 g) (see online Supplementary Table 1 for content). All selected participants were asked to maintain their diet and exercise routine throughout the study. Participants and the blood testing by technicians were blinded to the grouping status. Oil capsules provided to the two groups were produced to be consistent for appearance and taste to maintain the masking of the study participants.

Participants were randomized to take either n-3 PUFA oil capsules or corn oil capsules at the first visit (week 0) within 2 weeks of the screening visit. The second and third visits were at weeks 6 and 12 after the intervention, respectively. Physical measurement and clinical laboratory examination were conducted at each visit. A complete physical examination was conducted to measure the height, weight, waist circumference, hip circumference, and blood pressure on each participant at each visit. The body weight was measured while they were not wearing shoes or heavy clothing. The blood pressure was measured after the subjects had rested in a sitting position. Height and weight measurements were used to calculate the body mass index (BMI). Blood and fecal samples were also collected at each visit and were stored at −80°C until further laboratory examination. At the second and third visit, adverse event monitoring was undertaken by a brief interview based on questionnaire for recognized adverse symptoms of oil capsule supplements. Participants were asked to keep in touch with the researchers if any adverse effects were observed or if other drug treatment needed during the study period.

Quality control and compliance with the intervention protocol were insured among participants by requiring participants to maintain 3 day 24-h dietary recall (including two weekdays and one weekend day) before the intervention started, during the intervention at week 6, and before the intervention ended for 3 times, and to return any unconsumed capsules at the third visit. The fatty acid intake including the consumption from the capsules and dietary intake. Compliance was expressed as the number of capsules consumed (determined by the returned capsules) vs. the amount that would have been consumed with 100% compliance during the study period (>90%, excellent; 80–90%, good; <80%, poor). In addition, the plasma and erythrocyte membrane fatty acid composition were determined as a marker of compliance.

### Serum Sample Collection and Clinical Indicator Measurement

Fasting blood samples were drawn by a health care professional. The samples were immediately placed in an ice container and transferred to the Clinical Laboratory of Chronic Diseases Hospital in the Nanshan District. Blood samples were centrifuged at 3,000 rpm for 15 min at room temperature. The clinical indicators were detected by the automatic biochemical analyzer HITACH 7080.

### Fatty Acids Measurement

Plasma and red blood cells were obtained from whole blood EDTA samples and were stored at −80°C. Plasma samples were thawed at 4°C and mixed thoroughly before pretreatment. Red blood cell samples were mixed with hexane, centrifugated at 15,000 rpm for 3 min, and followed by removing supernatant to extract the erythrocyte membrane. The derivatization of fatty acids was achieved by the modified direct transesterification method proposed by Lepage (25). Fatty acid methyl esters were separated and analyzed by an Agilent 7890B gas chromatography coupled with an Agilent 5977A Series mass spectrometry with an Agilent DB-23 capillary column. The dilution series of the standard mixture of fatty acid methyl esters (Sigma Aldrich, Poole, UK) were used for the establishment of calibration curves. Individual fatty acids were presented as the % (w/w) content of the total fatty acid pool measured.

### Fecal Samples Collection and 16S Ribosomal RNA Gene Sequencing

Participants were instructed to collect their stool using MGIEasy stool sample collection kit (26). Uncontaminated samples were collected and then frozen at −80°C within 8 h. Microbial DNA extraction, PCR, and Illumina sequencing were performed as described previously (27). Briefly, DNA extraction was conducted using a QIAamp Fast DNA Stool Mini Kit (Qiagen, Valencia, CA, USA). The V3-V4 region of the bacteria's 16S ribosomal RNA (rRNA) gene was amplified with barcode-indexed primers (338F and 806R) by thermocycler Polymerase Chain Reaction system (GeneAmp 9700, ABI, Hampton, NH, USA) using FastPfu Polymerase. Amplicons were purified using AxyPrep DNA GelExtraction Kit (Axygen Biosciences, Union City, CA, USA), then quantified using QuantiFluor-ST (Promega, Madison, WI, USA). Sequencing was performed using the Illumina MiSeq platform (Illumina, San Diego, CA, USA).

### Fecal SCFA Measurement

Fecal samples for SCFA measurement were collected using aseptic swabs and sterile freeze storage tube. The details of fecal SCFA measurement have been described elsewhere (27). In short, SCFAs were measured with an Agilent 6890N gas chromatograph coupled with an Agilent 5975B mass spectrometer (Agilent Technologies Santa Clara, CA, USA). The calibration curves were obtained using internal standard quantitation was used for data acquisition and processing were conducted with the Varian MS workstation software (version 6.6).

## Statistical Analysis

General characteristics were summarized according to intervention group and control group as numbers (percentages) for categorical data, means ± standard deviations (SDs) for parametrically distributed data, and medians (interquartile range) for nonparametrically distributed data. Treatment effect was analyzed using the mixed linear model, with change from baseline as the response variable and supplementation (n-3 PUFAs enriched plant oil vs. corn oil) and time (weeks 0, 6, and 12) as the main effects. Within treatment changes for all variables were estimated by the least squares means technique within the mixed model. For the anthropometric indicators and clinical parameters, we conducted both an intent-to-treat (ITT) analysis and a per protocol (PP) analysis. All patients who met the inclusion criteria were included in the ITT analysis (*n* = 75) to maintain the original group composition achieved by randomization, and we carried the last observation forward for those time points where data were unavailable (28). For the PP analysis, subjects were included if they completed the intervention and all clinic visits, and were compliant with the intervention regimen (defined as consuming ≥ 80% of the oil capsules provided, consuming no additional food contained n-3 PUFA, and consuming no lipid-lowering drugs during the intervention period). Treatment differences in gut microbiota and metabolites were assessed from all available data, and we only conducted the PP analysis among the participants who provided fecal sample and completed the trial assignment to reflected the effects of the intervention unaffected by protocol deviations or non-adherence (29).

As stated above, participants who provided fecal samples at weeks 0, 6 and 12 were included in the 16S rRNA sequencing and further analysis. The 16S rRNA sequencing data were processed utilizing the Quantitative Insights Into Microbial Ecology platform (QIIME, Boulder, CO, USA, V.1.7.1) (30). Chimeras were removed and the operational taxonomic units (OTUs) were with 97% homology were generated using USEARCH (31). The identified taxonomy was aligned with Ribosomal Database Project Classifier (32) and Greengenes reference database (V.13.8). Alpha and beta diversity metrics were calculated in QIIME using the rarefied operational taxonomic unit (OTU) table. Beta diversity was assessed based on weighted UniFrac distances and permutational multivariate analysis of variance (PERMANOVA) with the use of the Adonis function in the “vegan” package^1^. For phylum- to genus-level microbiota groups, comparisons between intervention and control groups at the same time point were evaluated with Mann-Whitney test, within-group differences compared with the baseline were estimated with Wilcoxon matched-pair signed-rank test, between-group differences were calculated with a linear mixed model with package “lme4” (33). Enterotyping was conducted on a combined genus-abundance matrix of all samples on the basis of Jensen-Shannon divergence. Besides, the Calinski-Harabasz Index was utilized to determine the optimal number of the cluster. In the analysis of gut microbiota and predicted Kyoto Encyclopedia of Genes and Genomes (KEGG) biochemical pathways in each group, Wilcoxon matched-pair signed-rank test was adopted in each group. A two-tailed *P* < 0.05 was considered to be statistically significant. *P* values were corrected for multiple comparisons using the Benjamini-Hochberg procedure. Although no universal false discovery rate (FDR) significance threshold has been defined, a cut-point of 0.20 has been suggested (34) and used in the analysis of 16S rRNA sequencing studies (35, 36). Statistics were performed using R version 4.0.2 and SAS version 9.4 (SAS Institute).

## Results

### Subject Characteristics

We conducted screening questionnaires and blood lipid tests with 1,605 individuals and invited 255 potentially eligible participants to attend an in-person screening visit for informed consent. A total of 75 adults were willing to participate in the study and were randomly assigned to one of the two groups: 38 to the intervention group and 37 to the control group (Figure 1). All randomly assigned participants were included in the ITT analyses (*n* = 75). Twenty-four subjects were excluded from the PP analyses (*n* = 51): 5 dropped out for not being located, 10 dropped out for personal reasons and 9 were excluded for noncompliance. Of the included participants, 32% dropped out at different intervals throughout the study. The dropout rate was not significantly different between groups (*P* = 0.68). Neither of the two groups reported harms or unintended effects. The baseline characteristic of the subjects who completed the trial was shown in Table 1.

**Table 1:** Baseline characteristic of the participants completed the intervention.

	**Control group**	**Intervention group**
	**(***n*** = 25)**	**(***n*** = 26)**
Sex (male/female)	11/14	11/15
Age (years)^a^	53.16 ± 6.11	52.54 ± 8.85
Educational level (*n*)		
Senior high school or below	22	25
College or above	8	3
Current smoking (*n*)	4	3
Current drinking (*n*)	4	2
Weight (kg)^a^	63.60 ± 9.55	63.76 ± 12.31
BMI (kg/m^2^)^a^	24.98 ± 2.67	25.00 ± 3.04
Waist circumference (cm)^a^	87.58 ± 7.85	88.51 ± 9.06
Waist to hip ratio^a^	0.91 ± 0.11	0.91 ± 0.10
Diastolic pressure (mmHg)^a^	74.40 ± 11.34	73.54 ± 14.72
Systolic pressure (mmHg)^a^	119.68 ± 12.99	119.65 ± 20.27
TG (mmol/L)^a^	1.81 ± 1.55	2.05 ± 0.94
TC (mmol/L)^a^	5.60 ± 0.70	5.83 ± 1.09
LDL-C (mmol/L)	3.67 ± 0.56	3.86 ± 0.84
HDL-C (mmol/L)	1.37 ± 0.30	1.31 ± 0.24
ApoA1 (g/L)	1.76 ± 0.34	1.76 ± 0.29
ApoB (g/L)	1.08 ± 0.26	1.13 ± 0.31
Fasting glucose (mmol/L)	5.13 ± 0.56	5.16 ± 0.41
HbA1C (%)	5.70 ± 0.32	5.79 ± 0.34
Fasting insulin (μU/ml)	7.81 ± 3.39	9.45 ± 5.52

a *Values are Mean ± SD*.

### Energy and Macronutrient Intake

The mean energy intake of the control and intervention groups were 2050.6 (95% CI 1875.0 to 2226.2) kcal/day and 1977.8 (95% CI 1805.6 to 2150.0) kcal/day, respectively. Summary data for the macronutrient intakes were shown in Supplementary Table 2. There was no significant difference in the change of energy or macronutrient intake between two groups except for PUFAs. The mean change of PUFAs was 3.39 g/day (95% CI 1.71 to 5.07 g/day) for the intervention group and 0.35 g/day (95% CI−1.45 to 2.14 g/day) for the control diet. The relative increase for the intervention group was 3.04 g/day (95% CI 0.59 to 5.50 g/day, *P* = 0.02).

### Adherence to the Intervention

Participant's compliance to the intervention protocol was verified by the number of capsules consumed and fatty acid composition in plasma and erythrocyte membrane. The compliance was good in both two groups and the percentage of capsules consumed were not significantly different between groups (83.8% in the intervention group and 84.5% in the control group, *P* = 0.78). During the intervention, the concentrations of DPA in plasma as well as EPA in erythrocyte membrane were increased in the intervention group compared with the control group (*P* = 0.02 and *P* = 0.03, respectively). There was no significant treatment difference in the other fatty acids (Table 2).

**Table 2:** Changes in fatty acid composition of plasma and erythrocyte membrane in subjects at baseline, week 6 and week 12 of intervention^a^.

**Parameter**	**Change within group** ^**a**^						**Between groups^a^**	
	**Control group (*****n*** **= 25)**			**Intervention group (*****n*** **= 26)**			**Change**	***P*** **-value (PP)**
	**Baseline**	**Change week 6**	**Change week 12**	**Baseline**	**Change week 6**	**Change week 12**		
**Plasma**								
SFAs	47.49 (46.63, 48.35)	−0.19 (−0.76, 0.39)	−1.94 (−3.12,−0.76)**	46.46 (45.60, 47.32)	−0.02 (−0.52, 0.48)	−1.18 (−2.18,−0.17)*	0.76 (−0.79, 2.32)	0.33
MUFAs	13.24 (12.08, 14.41)	−0.22 (−1.32, 0.88)	0.61 (−1.13, 2.36)	14.45 (13.29, 15.62)	−0.82 (−1.78, 0.15)	−0.51 (−2.00, 0.97)	−1.13 (−3.42, 1.16)	0.33
PUFAs	39.27 (38.40, 40.14)	0.41 (−0.39, 1.20)	1.31 (0.32, 2.30)*	39.09 (38.22, 39.96)	0.84 (0.15, 1.53)*	1.69 (0.85, 2.53)***	0.38 (−0.92, 1.68)	0.56
ALA	0.30 (0.16, 0.44)	0.03 (−0.15, 0.21)	0.01 (−0.17, 0.20)	0.57 (0.43, 0.71)	0.09 (−0.07, 0.25)	−0.15 (−0.30, 0.01)	−0.16 (−0.40, 0.09)	0.19
EPA	0.49 (0.36, 0.62)	0.03 (−0.14, 0.20)	0.05 (−0.15, 0.24)	0.60 (0.47, 0.73)	0.13 (−0.02, 0.28)	0.19 (0.02, 0.36)*	0.14 (−0.12, 0.40)	0.28
DPA	0.46 (0.41, 0.52)	0.00 (−0.05, 0.06)	−0.02 (−0.11, 0.07)	0.44 (0.38, 0.49)	0.08 (0.03, 0.13)*	0.12 (0.05, 0.20)*	0.14 (0.03, 0.26)	0.02
DHA	4.20 (3.65, 4.75)	−0.10 (−0.48, 0.28)	−0.12 (−0.58, 0.33)	3.92 (3.38, 4.47)	−0.16 (−0.49, 0.18)	0.00 (−0.39, 0.38)	0.12 (−0.48, 0.72)	0.69
**Erythrocyte membrane**								
SFAs	46.68 (45.70, 47.65)	−1.06 (−2.16, 0.04)	−1.40 (−2.51,−0.30)	46.65 (45.70, 47.59)	−1.30 (−2.21,−0.39)	−1.49 (−2.40,−0.57)	−0.08 (−1.52, 1.36)	0.91
MUFAs	16.29 (15.58, 17.01)	−0.49 (−1.19, 0.21)	−0.97 (−1.84,−0.10)*	16.78 (16.10, 17.47)	−0.69 (−1.27,−0.10)*	−1.10 (−1.81,−0.39)**	−0.13 (−1.26, 1.00)	0.82
PUFAs	37.03 (35.59, 38.47)	1.55 (0.03, 3.06)	2.40 (0.68, 4.11)*	36.57 (35.18, 37.96)	1.99 (0.73, 3.25)*	2.58 (1.16, 4.00)***	0.19 (−2.04, 2.41)	0.87
ALA	0.86 (0.69, 1.04)	0.03 (−0.25, 0.31)	−0.10 (−0.32, 0.12)	1.05 (0.88, 1.22)	0.23 (0.00, 0.46)	0.12 (−0.06, 0.30)	0.22 (−0.06, 0.51)	0.12
EPA	0.33 (0.27, 0.40)	0.00 (−0.05, 0.06)	0.02 (−0.05, 0.08)	0.31 (0.24, 0.37)	0.07 (0.02, 0.11)**	0.11 (0.06, 0.17)***	0.10 (0.01, 0.19)	0.03
DPA	0.95 (0.84, 1.05)	−0.05 (−0.16, 0.06)	−0.02 (−0.16, 0.11)	0.97 (0.87, 1.08)	0.00 (−0.09, 0.09)	0.06 (−0.05, 0.17)	0.08 (−0.09, 0.26)	0.34
DHA	5.46 (4.84, 6.07)	−0.08 (−0.42, 0.25)	0.07 (−0.31, 0.44)	5.20 (4.60, 5.80)	−0.08 (−0.36, 0.20)	0.07 (−0.24, 0.38)	0.00 (−0.49, 0.48)	0.99

Data are mean (lower confidence limit, upper confidence limit). Fatty acid composition of plasma and erythrocyte membrane were obtained at week 0, representing baseline, and weeks 6 and 12 - baseline, representing change from baseline. Significant difference from baseline (*, P < 0.05; **, P < 0.01; ***, P < 0.001).

a *Mean, confidence limits, and P-values determined using repeated-measures least squares means in PROC MIXED of SAS 9.4 with all available data*.

### Changes in Anthropometric and Clinical Indicators

The supplementation of plant-derived n-3 PUFA significantly decreased TC concentrations compared with the control group (*P* < 0.05) (Table 3). During the intervention, the mean TC change was −0.48 mmol/L (95% CI−0.78 to−0.19 mmol/L, *P* < 0.01) for the intervention group and−0.06 mmol/L (95% CI−0.36 to 0.24 mmol/L, *P* = 0.24) for the control diet. The relative change for the intervention group was−0.43 mmol/L (95% CI−0.84 to−0.01 mmol/L, *P* = 0.02). There was no significant treatment difference in anthropometric and clinical indicators such as weight, BMI, waist circumference, waist to hip ratio, blood pressure, fasting TG, HDL-C, ApoA1, ApoB, glucose, HbA1C, and insulin. ITT analysis produced similar results to the PP analysis with respect to significant findings (Table 3).

**Table 3:** Changes from baseline in anthropometric markers and clinical indicators in subjects at baseline and following oil capsule intervention*^a^*.

**Parameter**	**Changes within group^a^**						**Between groups^a^**		
	**Control group (*****n*** **= 25)**			**Intervention group (n = 26)**			**Changes**	***P*** **-value (PP)**	***P*** **-value (ITT) ^b^**
	**Baseline**	**Changes in week 6**	**Changes in week 12**	**Baseline**	**Changes in week 6**	**Changes in week 12**			
**Anthropometric markers**									
Weight (kg)	63.60 (59.16, 68.04)	0.08 (−0.45, 0.60)	0.59 (−0.02, 1.19)	63.76 (59.40, 68.11)	−0.52 (−1.03, 0.00)	0.13 (−0.46, 0.71)	−0.46 (−1.30, 0.38)	0.28	0.27
BMI (kg/m^2^)	24.98 (23.83, 26.14)	0.01 (−0.28, 0.30)	0.15 (−0.11, 0.41)	25.00 (23.87, 26.13)	0.04 (−0.25, 0.32)	0.13 (−0.12, 0.38)	−0.03 (−0.39, 0.33)	0.89	0.87
WC (cm)	87.58 (84.17, 91.00)	−0.30 (−2.67, 2.07)	0.68 (−1.72, 3.08)	88.51 (85.16, 91.85)	0.81 (−1.52, 3.13)	1.53 (−0.80, 3.87)	0.85 (−2.49, 4.20)	0.61	0.62
Waist to hip ratio	0.91 (0.87, 0.95)	−0.02 (−0.06, 0.02)	−0.01 (−0.06, 0.04)	0.91 (0.87, 0.95)	0.01 (−0.03, 0.05)	0.00 (−0.04, 0.05)	0.01 (−0.05, 0.08)	0.66	0.65
SP (mmHg)	119.68 (112.81, 126.55)	−0.68 (−5.29, 3.93)	2.77 (−4.28, 9.81)	119.65 (112.92, 126.39)	−1.08 (−5.60, 3.45)	−1.46 (−8.25, 5.33)	−4.23 (−14.01, 5.55)	0.39	0.39
DP (mmHg)	74.40 (69.11, 79.69)	1.00 (−2.07, 4.07)	3.19 (−0.87, 7.24)	73.54 (68.35, 78.73)	0.12 (−2.89, 3.13)	2.42 (−1.48, 6.33)	−0.76 (−6.39, 4.87)	0.79	0.76
**Clinical indicators**									
TG (mmol/L)	1.81 (1.29, 2.32)	−0.17 (-0.47, 0.13)	−0.36 (−0.68,−0.05)*	2.05 (1.54, 2.55)	−0.28 (−0.57, 0.02)	−0.52 (−0.83,−0.21)**	−0.15 (−0.60, 0.29)	0.49	0.56
TC (mmol/L)	5.60 (5.23, 5.97)	0.08 (−0.13, 0.29)	−0.06 (−0.36, 0.24)	5.83 (5.47, 6.20)	−0.29 (−0.50,−0.09)**	−0.48 (−0.78,−0.19)**	−0.43 (−0.84, −0.01)	<0.05	<0.05
LDL-C (mmol/L)	3.67 (3.38, 3.95)	0.12 (−0.05, 0.29)	0.08 (−0.13, 0.29)	3.86 (3.58, 4.14)	−0.16 (−0.32, 0.01)	−0.20 (−0.40, 0.01)	−0.28 (−0.58, 0.02)	0.07	0.07
HDL-C (mmol/L)	1.37 (1.26, 1.48)	−0.01 (−0.05, 0.03)	−0.02 (−0.09, 0.04)	1.31 (1.20, 1.42)	−0.05 (−0.08,−0.01)*	−0.08 (−0.14, −0.02)*	−0.05 (−0.14, 0.03)	0.24	0.26
ApoA1 (g/L)	1.76 (1.63, 1.88)	0.07 (−0.02, 0.16)	0.09 (0.00, 0.18)	1.76 (1.63, 1.88)	−0.03 (−0.12, 0.06)	0.00 (−0.09, 0.09)	−0.09 (−0.22, 0.04)	0.17	0.16
ApoB (g/L)	1.08 (0.97, 1.20)	0.07 (0.00, 0.14)	0.07 (−0.02, 0.16)	1.13 (1.02, 1.25)	−0.03 (−0.10, 0.04)	−0.02 (−0.10, 0.07)	−0.09 (−0.21, 0.03)	0.16	0.15
FPG (mmol/L)	5.13 (4.93, 5.33)	−0.25 (−0.40,−0.09)*	−0.05 (−0.18, 0.08)	5.16 (4.96, 5.35)	−0.25 (−0.40,−0.10)*	−0.06 (-0.18, 0.07)	0.00 (−0.18, 0.17)	0.96	0.98
HbA1C (%)	5.70 (5.56, 5.83)	0.03 (−0.02, 0.08)	0.02 (−0.03, 0.08)	5.79 (5.66, 5.92)	0.03 (−0.02, 0.09)	0.05 (−0.01, 0.10)	0.02 (−0.05, 0.10)	0.55	0.57
FPI (μU/ml)	7.81 (5.96, 9.66)	−0.28 (−1.67, 1.11)	0.26 (−1.67, 2.18)	9.45 (7.64, 11.26)	0.43 (−0.94, 1.79)	−0.10 (−1.99, 1.79)	−0.35 (−3.05, 2.35)	0.79	0.79

*Data are mean (lower confidence limit, upper confidence limit). Anthropometric and clinical measures were obtained at week 0, representing baseline, and weeks 6 and 12 - baseline, representing change from baseline*.

* Significant difference from baseline (*, P < 0.05; **, P < 0.01).

a *Mean, confidence limits, and P-values determined using repeated-measures least squares means in PROC MIXED of SAS 9.4 with all available data*.

b *All patients who met the inclusion criteria were included in the ITT analysis (n = 75), and the last observations were carried forward for those time points where data were unavailable. WC, waist circumference; SP, systolic pressure; DP, diastolic pressure; PFG, fasting plasma glucose; FPI, fasting plasma insulin*.

### Changes in the Intestinal Microbiota

**Figure 2:**
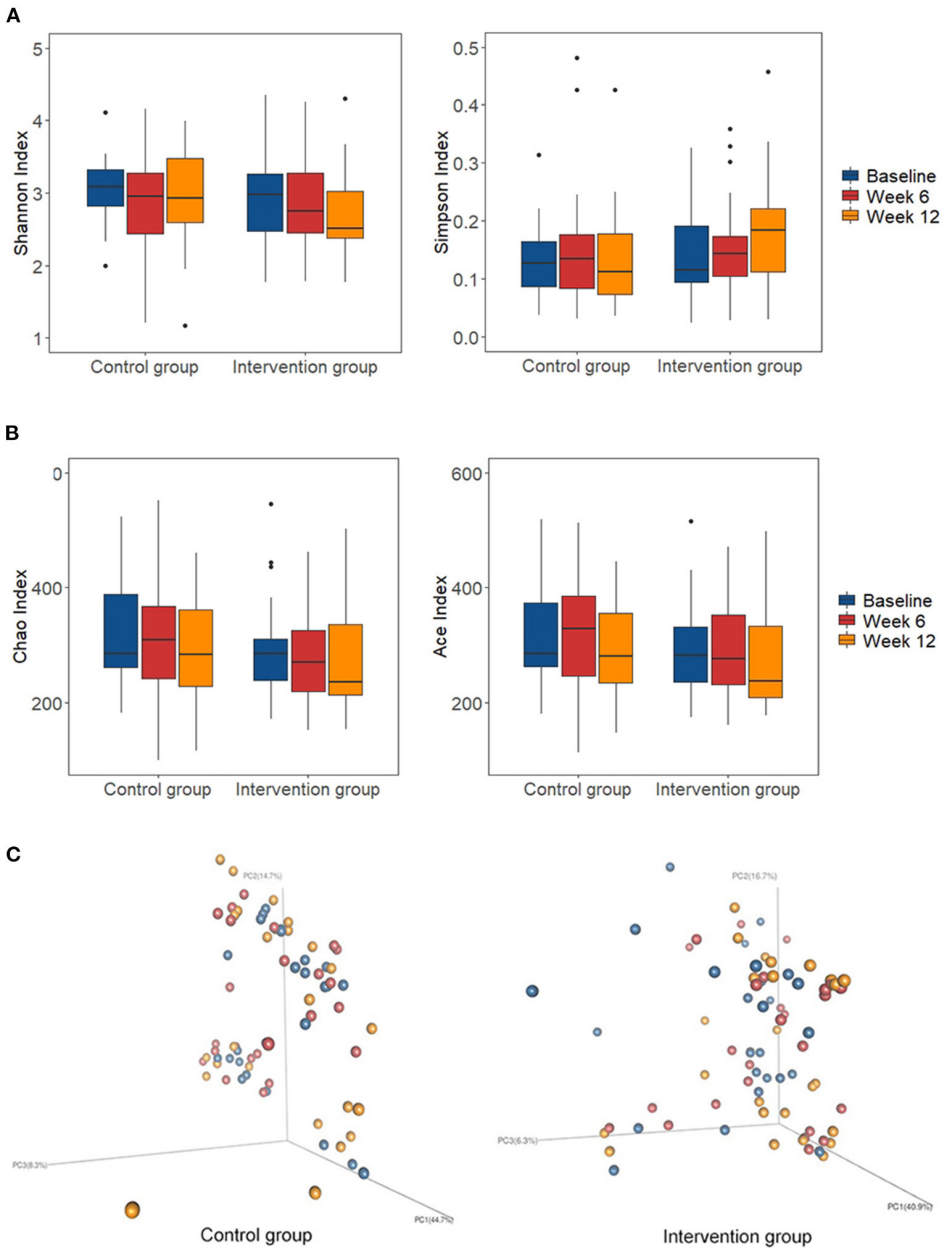
Changes of global gut microbiota after intervention in each group. **(A)** The microbial community diversity estimated by Shannon and Simpson estimator at OTU level; **(B)** The microbial community richness estimated by Chao and Ace estimator at OTU level; **(C)** Principal coordinate analysis (PCoA) score plots based on weighted Unifrac analysis at OTU level in the control and intervention group at baseline (blue), week 6 (red), and week 12 (orange). Box represents the interquartile range, the line inside represents the median, whiskers represent 10-90 percentiles, dot represents outliers that are past the ends of the whiskers.

The microbial community diversity indicated by the Shannon diversity index and Simpson diversity index (Figure 2A), and the community richness estimated by the Ace and Chao1 estimators (Figure 2B) showed no significant change in the two groups during the intervention. The two groups showed no significant shift in the overall composition of the gut microbiota at OTU levels as indicated by PERMANOVA analysis (*P* > 0.05 for both) (Figure 2C). The enterotypes remained stable in both of the two groups (Supplementary Figure 1).

**Figure 3:**
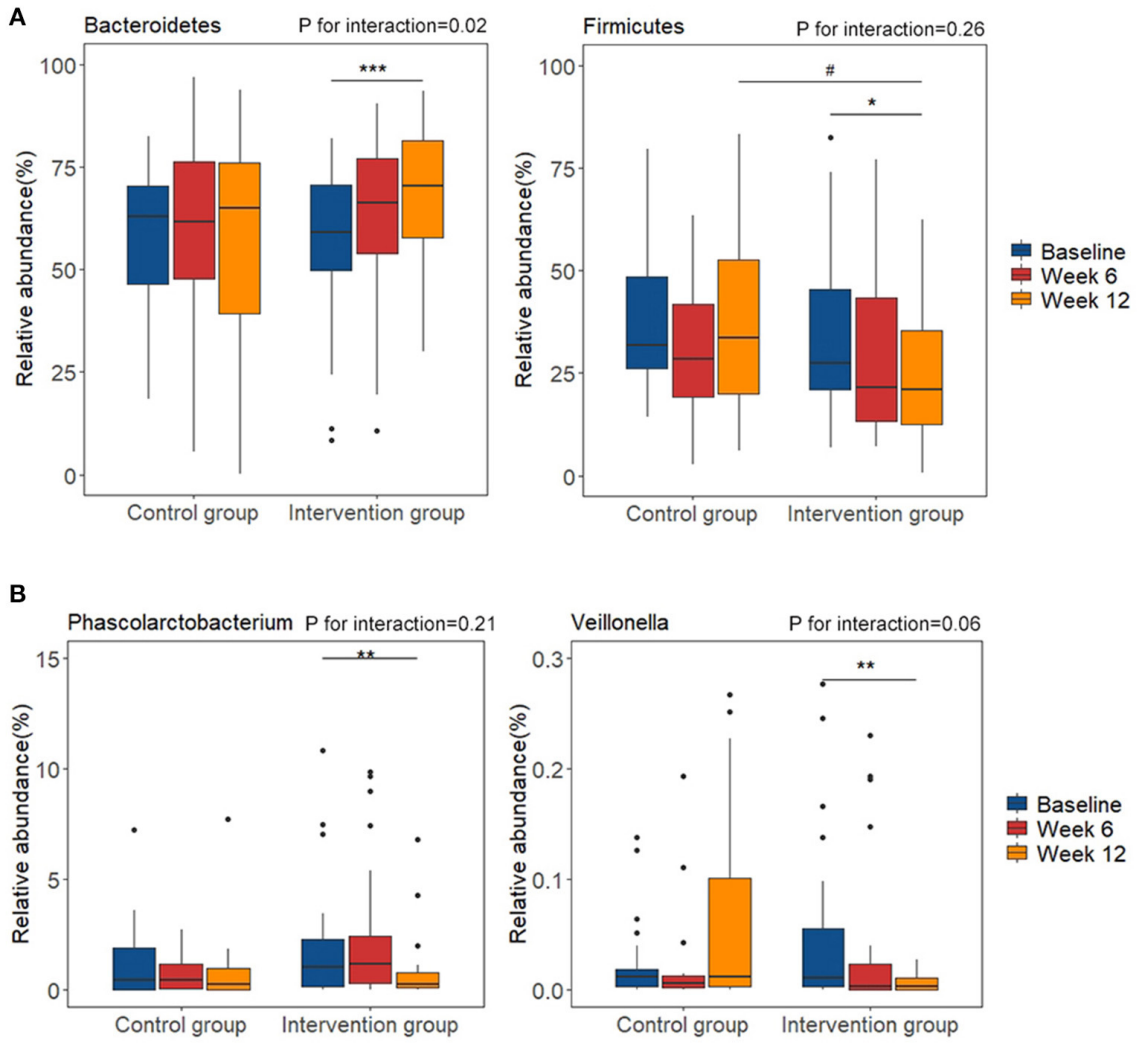
Changes of relative abundance of gut microbiota composition before and after diet intervention. **(A)** phylum level; **(B)** genus level. *P < 0.05, **P < 0.01, ***P < 0.001 vs. baseline of the same group. ^#^*P* < 0.05 vs. control group at the same time point.

At phylum level, the relative abundance of *Bacteroidetes* significantly increased in the intervention group after intervention (*P* < 0.01; FDR corrected *p* = 0.11), while the relative abundance of *Firmicutes* was marginally decreased (*P* < 0.05; FDR corrected *p* = 0.23) (Figure 3A). We then calculated the ratio between *Firmicutes* and *Bacteroidetes* (F/B ratio), which widely used as a marker of gut dysbiosis and found it to be significantly decreased in the intervention group after intervention (*P* < 0.05; FDR corrected *p* = 0.16). Compared with the control group, the abundance of the *Firmicutes* was significantly lower in the intervention group after intervention (*P* < 0.05; FDR corrected *p* = 0.18). Between-group analysis highlighted the significant interaction of n-3 PUFA supplementation and groups (time^*^group) on *Bacteroidetes* (*P* for interaction = 0.02; FDR corrected *p* = 0.15) and F/B ratio (*P* for interaction = 0.04; FDR corrected *p* = 0.15). At genus level, the intervention of plant-derived n-3 PUFAs resulted in a significant decrease in relative abundance of *Phascolarctobacterium* (*P* < 0.01; FDR corrected p = 0.18) and *Veillonella* (*P* < 0.01; FDR corrected *p* = 0.18) after the intervention (Figure 3B). The changes during the intervention at class, order, and family levels were shown in Supplementary Figure 2. The relative abundance of *Bacteroidia* and *Bacteroidales* were increased, and the relative abundance of *Bacilli, Negativicutes, Enterobacteriales, Selenomonadales, Veillonellaceae, Acidaminococcaceae*, and *Streptococcaceae* were decreased (FDR corrected *p* < 0.20 for all). Between-group analysis showed the significant interaction of n-3 PUFA supplementation and groups on *Bacteroidia* (*P* for interaction = 0.02; FDR corrected *p* = 0.15), *Bacilli* (*P* for interaction < 0.01; FDR corrected *p* = 0.08), *Bacteroidales* (*P* for interaction = 0.02; FDR corrected *p* = 0.18).

**Figure 4:**
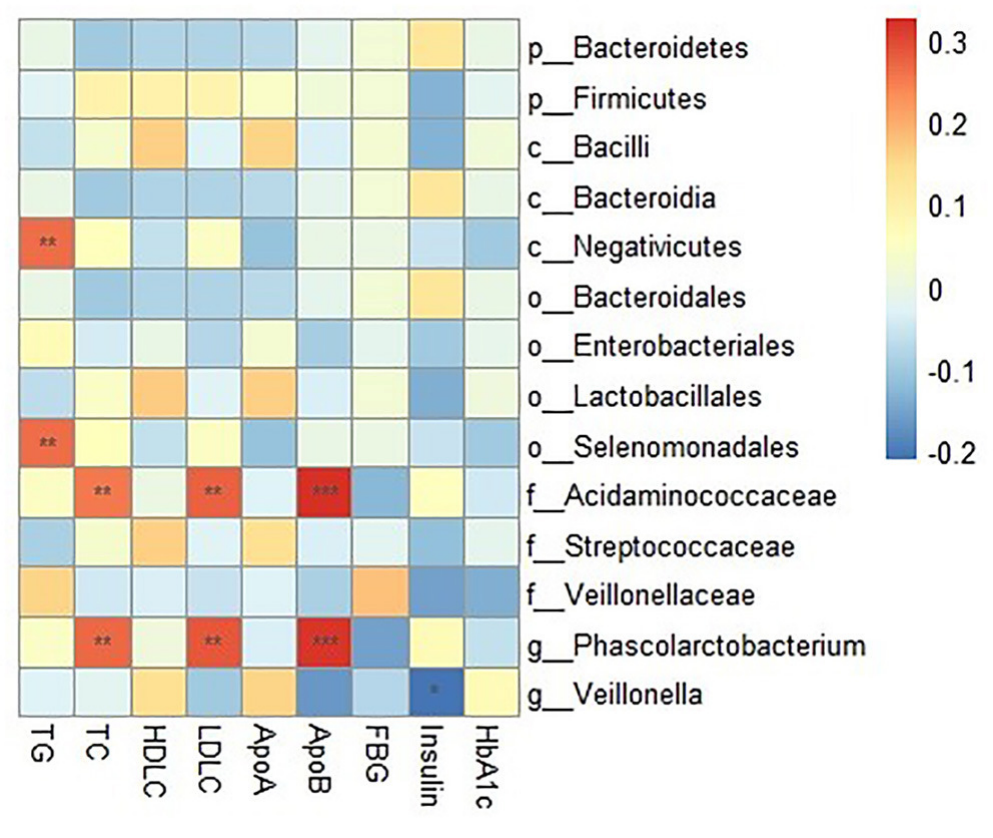
Correlation between clinical indicators and relative abundance of gut microbiota composition. The intensity of the colors represents the degree of association between the concentration of clinical indicators and relative abundance of gut microbiota composition as measured by the Spearman's correlations. *P*-values were corrected for multiple testing using the Benjamini-Hochberg false discovery rate. *P < 0.05, **P < 0.01, ***P < 0.001.

The relative abundance of *Negativicutes* and *Selenomonadales* were positively associated with TG in the correlation analysis (*r* = 0.26, FDR corrected *p* < 0.05 for all). The relative abundance of *Acidaminococcaceae* was positively associated with TC (*r* = 0.25, FDR corrected *p* < 0.05), LDL-C (*r* = 0.27, FDR corrected *p* < 0.05) and ApoB (*r* = 0.32, FDR corrected *p* < 0.01). And the relative abundance of *Phascolarctobacterium* was positively correlated with three blood lipid indicators (r = 0.27, FDR corrected *p* < 0.05 for TC; *r* = 0.28, FDR corrected *p* < 0.05 for LDL-C and *r* = 0.31, FDR corrected *p* < 0.01 for ApoB, respectively) (Figure 4).

No significant changes in KEGG database biochemical pathways were seen in the control group. In the intervention group, 22 pathways were identified as significantly responding to the plant-derived n-3 PUFAs intervention after FDR correction (FDR corrected *p* < 0.20) (Supplementary Table 3). Functional prediction analysis indicated that genes involved in energy, amino acid, and carbohydrate metabolism likely exhibited enrichment.

### Changes in Fecal SCFAs

Fecal acetic acid, propionic acid, butyric acid, isobutyric acid, valeric acid, isovaleric acid, and total SCFAs concentration did not significantly differ within and between the groups. There was no significant correlation between the relative abundance of gut microbiota and the fecal SCFAs (data not shown).

## Discussion

In this randomized controlled trial in subjects with marginal hyperlipidemia, increased DPA concentrations in plasma and EPA concentrations in erythrocyte phospholipids were observed in the intervention group compared with the control group, which providing unbiased support for subject compliance to the protocol. Thus, ALA/SDA-rich natural oils have the potential to provide a useful plant-derived dietary resource for increasing tissue concentrations of long-chain n-3 fatty acids in humans (37). A large proportion of scientific research has reported the beneficial effects of ALA in many diseases (6, 38, 39). However, the effects of ALA on blood lipid profiles were controversial in recent decades (10, 11), and existing studies about the beneficial effect of SDA were still limited (9). In the current study, plant-derived n-3 PUFA supplementation significantly decreased the TC concentration in subjects with marginal hyperlipidemia after the 12-week intervention. Moreover, TG concentration significantly decreased at week 12 compared with baseline in the intervention group and marginal treatment effect was observed for the LDL-C concentration. Despite the improved trends were showed, no significant difference between groups was observed for TG and LDL-C, which may due to the relative short intervention period of our study. Our results were in accordance with a most recent meta-analysis which indicated that supplementation with ALA could significantly reduce the concentrations of TC and other lipid indicators (12). Despite our intervention dose was lower than the dose of maximum lipid-lowering effect with 3–8 g ALA per day elucidated in that meta-analysis, the beneficial effect of the plant-derived n-3 PUFAs on serum levels of TC was still observed in our study. This may be due to the following explanations: first, only subjects with a low daily intake of n-3 PUFAs were included to avoid the potential “ceiling effect” (40); second, the combination of ALA and SDA may be more effective than single ALA owing to the more efficient conversion of SDA to EPA, which was affected by the rate-limiting activity of Δ6-desaturase (37, 41); Third, the effects of ALA on the reduction of serum lipid concentration in Asian countries were more obvious than that in American or European countries (12). More high-quality randomized controlled trials are needed in the future to validate the results of our study.

The disturbance of lipid metabolism could induce changes the intestinal environment and further leading to the dysbiosis of internal microflora (42). A recent review summarized that individuals with hyperlipidemia exhibit disturbed structure and function of gut microbiota, such as lower abundance of *Akkermansia, Bacteroides, Roseburia*, and *Faecalibacterium* (43). In our result, plant-derived n-3 PUFA supplementation decreased the F/B ratio in subjects with marginal hyperlipidemia. The F/B ratio has been widely accepted to have an important influence in maintaining normal intestinal homeostasis. Previous studies showed that obesity and hyperlipidemia were associated with an increased ratio of F/B (44, 45). Our results were in accordance with studies in animal models which found that plant-derived n-3 PUFA could reduce the abnormal ratio of F/B and improve gut dysbiosis (46, 47). Despite the gut dysbiosis varies across diseases, the common manifestation was a proportional increase in facultative anaerobes and a decrease in the typical anaerobes that dominate the adult healthy gut (48). In our study, several facultative anaerobes, such as *Bacilli, Streptococcaceae* and *Enterobacteriales*, were significantly decreased, and several typical anaerobes, such as *Bacteroidia* and *Bacteroidales*, were significantly increased after the intervention. The imbalance in microbial communities linked with hyperlipidemia seems to be corrected by plant-derived n-3 PUFA supplementation. Initial evidence showed that decreasing F/B ratio was associated with an increased production of SCFAs (20). However, fecal SCFA concentrations did not significantly differ within and between the groups in the current study, which may be due to the conversion among SCFAs through microbial cross-feeding (49) and the metabolism to supply energy for colonocytes (50). In addition, although subjects were required to maintain their dietary habits during the intervention, intra-individual changes in undigested carbohydrate intake may have a more direct effect on SCFAs than n-3 PUFA supplementation.

At genus level, *Phascolarctobacterium* was found to significantly decreased after the n-3 PUFA intervention, and the relative abundance of *Phascolarctobacterium* was positively associated with blood lipids such as TC, and LDL-C levels. *Phascolarctobacterium* (family: *Acidaminococcaceae*; order: *Selenomonadales*; class: *Negativicutes*; phylum: *Firmicutes*) was a known propionate-producing bacterium (51) and closely associated with carbohydrate intake (52–54). *Phascolarctobacterium* has also been previously found to be negatively correlated with the intake of PUFAs in the diet (55). A high abundance of *Phascolarctobacterium* was found to be associated with high insulin sensitivity (56), and a reduction in systemic inflammation (57). Although *Phascolarctobacterium* seems to be generally health-promoting, it is common that a gut bacterium associated with health in one setting could be associated with disease in another context. For example, *Phascolarctobacterium* was observed to increase in the process of prostate cancer (58) and be enriched in patients with tuberculosis (51). In our study, another gut microbiota, *Veillonella*, was also found to significantly decreased after the n-3 PUFA intervention. In a previous study, *Veillonella* (family: *Veillonellaceae*; order: *Selenomonadales*; class: *Negativicutes*; phylum: *Firmicutes*) was associated with higher levels of gut inflammation, and the development of colitis (59). Significantly higher *Veillonella* has been observed in subjects with gestational diabetes and hyperlipidemia, and the relative abundance of *Veillonella* was positively correlated to TC levels (60). Given that the serum levels of TC decreased during the intervention in our study, it is possible that the favorable effects of the plant-derived n-3 PUFA in our study might be due to the influence on gut microbiota.

There are several strengths of the current study. First, screening patients with a low daily intake of n-3 PUFAs for intervention could avoid the “ceiling effect.” Second, the patients were suggested to maintain their usual diet and the diet habitual during the intervention was monitored to make sure that the existing eating habits were stable. Third, strict exclusion criteria ensured that none of the participants took medications for hyperlipidemia or other chronic metabolic diseases, which could avoid the potential confounding from the usage of medication and their influence on gut microbiota (61, 62). Our study also has certain limitations. First, the 12-week intervention was still too short for us to examine the long-term effect of n-3 PUFA supplements on gut microbiota and their metabolites. Second, the moderately high dropout rates in the current study could result in confounding and selection bias. However, the ITT and PP analysis produced similar results with respect to significant findings. Third, we did not compare the diversity of gut microbiota between healthy and hyperlipidemia individuals, so it is not possible to determine whether the changes in microbiota abundance that occurred during the intervention were ameliorating the microbiota disturbance caused by the morbid state.

## Conclusion

In summary, plant-derived n-3 PUFAs beneficially affect the serum level of TC during the 12-week intervention period. Moreover, plant-derived n-3 PUFA supplementation decreased the F/B ratio, decreased the relative abundance of facultative anaerobes, and increased the typical anaerobes, thus improving gut dysbiosis.

## Data Availability Statement

The datasets presented in this study can be found in online repositories. The names of the repository/repositories and accession number(s) can be found at: https://www.ncbi.nlm.nih.gov/sra/PRJNA796472.

## Ethics Statement

The studies involving human participants were reviewed and approved by Tongji Medical College. The patients/participants provided their written informed consent to participate in this study.

## Author Contributions

HL: formal analysis, data curation, and writing-original draft. XL: formal analysis, writing-review and editing, and methodology. YZ: formal analysis and data curation. YH, QZ, SL, CF, LLi, and YL: data curation. WM: revising-original draft. XP: conceptualization and revising-original draft. JY: formal analysis, methodology, revising-original draft, and supervision. LLiu: conceptualization and supervision. All authors contributed to the article and approved the submitted version.

## Funding

This study was supported by Grant NSFC81820108027 from the Major International (Regional) Joint Research Project of the National Natural Science Foundation of China; Grant 2017YFC1600500 from the National Key Research and Development Program of China; and Grant 82103832 from the Young Scientists Fund of the National Natural Science Foundation of China. The funders had no role in the design and conduct of the study; collection, management, analysis, and interpretation of the data; preparation, review, or approval of the manuscript; and decision to submit the manuscript for publication.

## Conflict of Interest

The authors declare that the research was conducted in the absence of any commercial or financial relationships that could be construed as a potential conflict of interest.

## Publisher's Note

All claims expressed in this article are solely those of the authors and do not necessarily represent those of their affiliated organizations, or those of the publisher, the editors and the reviewers. Any product that may be evaluated in this article, or claim that may be made by its manufacturer, is not guaranteed or endorsed by the publisher.

## Footnotes

^1^Available online at: https://rweb.stat.umn.edu/R/site-library/vegan/html/CCorA.html

## References

[1] Kopin L Lowenstein C. Dyslipidemia. Ann Intern Med. (2017) 167:Itc81–96. 10.7326/AITC201712050 29204622

[2] Opoku S Gan Y Fu W Chen D Addo-Yobo E Trofimovitch D. Prevalence and risk factors for dyslipidemia among adults in rural and urban China: findings from the China national stroke screening and prevention project (CNSSPP). BMC Public Health. (2019) 19:1500. 10.1186/s12889-019-7827-5 31711454 PMC6849283

[3] Navar-Boggan AM Peterson ED D'Agostino RB Neely B Sniderman AD Pencina MJ. Hyperlipidemia in early adulthood increases long-term risk of coronary heart disease. Circulation. (2015) 131:451–8. 10.1161/CIRCULATIONAHA.114.012477 25623155 PMC4370230

[4] Chiavaroli L Nishi SK Khan TA Braunstein CR Glenn AJ Mejia SB. Portfolio dietary pattern and cardiovascular disease: a systematic review and meta-analysis of controlled trials. Prog Cardiovasc Dis. (2018) 61:43–53. 10.1016/j.pcad.2018.05.004 29807048

[5] Wu JHY Micha R Mozaffarian D. Dietary fats and cardiometabolic disease: mechanisms and effects on risk factors and outcomes. Nat Rev Cardiol. (2019) 16:581–601. 10.1038/s41569-019-0206-1 31097791

[6] Pan A Chen M Chowdhury R Wu JH Sun Q Campos H. alpha-Linolenic acid and risk of cardiovascular disease: a systematic review and meta-analysis. Am J Clin Nutr. (2012) 96:1262–73. 10.3945/ajcn.112.044040 23076616 PMC3497923

[7] Del Gobbo LC Imamura F Aslibekyan S Marklund M Virtanen JK Wennberg M. omega-3 Polyunsaturated fatty acid biomarkers and coronary heart disease: pooling project of 19 cohort studies. JAMA Intern Med. (2016) 176:1155–66. 10.1001/jamainternmed.2016.2925 27357102 PMC5183535

[8] Ursin VM. Modification of plant lipids for human health: development of functional land-based omega-3 fatty acids. J Nutr. (2003) 133:4271–4. 10.1093/jn/133.12.4271 14652387

[9] Baker EJ Miles EA Burdge GC Yaqoob P Calder PC. Metabolism and functional effects of plant-derived omega-3 fatty acids in humans. Prog Lipid Res. (2016) 64:30–56. 10.1016/j.plipres.2016.07.002 27496755

[10] Wendland E Farmer A Glasziou P Neil A. Effect of alpha linolenic acid on cardiovascular risk markers: a systematic review. Heart. (2006) 92:166–9. 10.1136/hrt.2004.053538 15890766 PMC1860766

[11] Balk EM Lichtenstein AH Chung M Kupelnick B Chew P Lau J. Effects of omega-3 fatty acids on serum markers of cardiovascular disease risk: a systematic review. Atherosclerosis. (2006) 189:19–30. 10.1016/j.atherosclerosis.2006.02.012 16530201

[12] Yue H Qiu B Jia M Liu W Guo XF Li N. Effects of α-linolenic acid intake on blood lipid profiles:a systematic review and meta-analysis of randomized controlled trials. Crit Rev Food Sci Nutr. (2020) 9:1–17. 10.1080/10408398.2020.1790496 32643951

[13] Kuhnt K Weiß S Kiehntopf M Jahreis G. Consumption of echium oil increases EPA and DPA in blood fractions more efficiently compared to linseed oil in humans. Lipids Health Dis. (2016) 15:32. 10.1186/s12944-016-0199-2 26892399 PMC4757976

[14] Pieters D Mensink R. Effects of stearidonic acid on serum triacylglycerol concentrations in overweight and obese subjects: a randomized controlled trial. Eur J Clin Nutr. (2015) 69:121–6. 10.1038/ejcn.2014.193 25226826

[15] Surette ME Edens M Chilton FH Tramposch KM. Dietary echium oil increases plasma and neutrophil long-chain (n-3) fatty acids and lowers serum triacylglycerols in hypertriglyceridemic humans. J Nutr. (2004) 134:1406–11. 10.1093/jn/134.6.1406 15173404

[16] Witkowski M Weeks TL Hazen SL. Gut microbiota and cardiovascular disease. Circ Res. (2020) 127:553–70. 10.1161/CIRCRESAHA.120.316242 32762536 PMC7416843

[17] Tang WH Kitai T Hazen SL. Gut microbiota in cardiovascular health and disease. Circ Res. (2017) 120:1183–96. 10.1161/CIRCRESAHA.117.309715 28360349 PMC5390330

[18] Fu J Bonder MJ Cenit MC Tigchelaar EF Maatman A Dekens JA. The gut microbiome contributes to a substantial proportion of the variation in blood lipids. Circ Res. (2015) 117:817–24. 10.1161/CIRCRESAHA.115.306807 26358192 PMC4596485

[19] Granado-Serrano AB Martin-Gari M. Faecal bacterial and short-chain fatty acids signature in hypercholesterolemia. Sci Rep. (2019) 9:1772. 10.1038/s41598-019-38874-3 30742005 PMC6370822

[20] Costantini L Molinari R. Impact of omega-3 fatty acids on the gut microbiota. Int J Mol Sci. (2017) 18:2645. 10.3390/ijms18122645 29215589 PMC5751248

[21] Tindall AM McLimans CJ Petersen KS Kris-Etherton PM Lamendella R. Walnuts and vegetable oils containing oleic acid differentially affect the gut microbiota and associations with cardiovascular risk factors: follow-up of a randomized, controlled, feeding trial in adults at risk for cardiovascular disease. J Nutr. (2020) 150:806–17. 10.1093/jn/nxz289 31848609 PMC7138683

[22] Todorov H Kollar B Bayer F Brandão I Mann A Mohr J. α-Linolenic Acid-Rich Diet Influences Microbiota Composition and Villus Morphology of the Mouse Small Intestine. Nutrients. (2020) 12:732. 10.3390/nu12030732 32168729 PMC7146139

[23] Segura Munoz RR Quach T Gomes-Neto JC Xian Y Pena PA Weier S. Stearidonic-enriched soybean oil modulates obesity, glucose metabolism, and fatty acid profiles independently of akkermansia muciniphila. Mol Nutr Food Res. (2020) 64:e2000162. 10.1002/mnfr.202000162 32656952 PMC8606245

[24] [Chinese guidelines on prevention and treatment of dyslipidemia in adults]. Zhonghua Xin Xue Guan Bing Za Zhi. (2007) 35:390–419. 10.3760/j.issn:0253-3758.2007.05.003 17711682

[25] Lepage G Roy CC. Direct transesterification of all classes of lipids in a one-step reaction. J Lipid Res. (1986) 27:114–20. 10.1016/S0022-2275(20)38861-1 3958609

[26] Han M Hao L Lin Y Li F Wang J Yang H. A novel affordable reagent for room temperature storage and transport of fecal samples for metagenomic analyses. Microbiome. (2018) 6:43. 10.1186/s40168-018-0429-0 29482661 PMC5828344

[27] Li X Yin J Zhu Y Wang X Hu X Bao W. Effects of whole milk supplementation on gut microbiota and cardiometabolic biomarkers in subjects with and without lactose malabsorption. Nutrients. (2018) 10:1403. 10.3390/nu10101403 30279333 PMC6213503

[28] Sedgwick P. Intention to treat analysis versus per protocol analysis of trial data. BMJ. (2015) 350:h681. 10.1136/bmj.h681 25663096

[29] Tripepi G Chesnaye NC Dekker FW Zoccali C Jager KJ. Intention to treat and per protocol analysis in clinical trials. Nephrology. (2020) 25:513–7. 10.1111/nep.13709 32147926

[30] Kuczynski J Stombaugh J Walters WA González A Caporaso JG Knight R. Using QIIME to analyze 16S rRNA gene sequences from microbial communities. Curr Protoc Bioinformatics. Chapter: Unit 10.7 (2011). 10.1002/0471250953.bi1007s36 22161565 PMC3249058

[31] Edgar RC. Search and clustering orders of magnitude faster than BLAST. Bioinformatics. (2010) 26:2460–1. 10.1093/bioinformatics/btq461 20709691

[32] Cole JR Wang Q Cardenas E Fish J Chai B Farris RJ. The ribosomal database project: improved alignments and new tools for rRNA analysis. Nucleic Acids Res. (2009) 37:D141–5. 10.1093/nar/gkn879 19004872 PMC2686447

[33] Bates D. Fitting linear mixed-effects models using the lme4 package in R. J Stat Softw. (2014) 67:1–48. 10.18637/jss.v067.i01

[34] Smith NL Hindorff LA Heckbert SR Lemaitre RN Marciante KD Rice K. Association of genetic variations with nonfatal venous thrombosis in postmenopausal women. JAMA. (2007) 297:489–98. 10.1001/jama.297.5.489 17284699

[35] Gershuni V Li Y Elovitz M Li H Wu GD Compher CW. Maternal gut microbiota reflecting poor diet quality is associated with spontaneous preterm birth in a prospective cohort study. Am J Clin Nutr. (2021) 113:602–11. 10.1093/ajcn/nqaa361 33515003 PMC7948858

[36] Nicolucci AC Hume MP Martínez I Mayengbam S Walter J Reimer RA. Prebiotics reduce body fat and alter intestinal microbiota in children who are overweight or with obesity. Gastroenterology. (2017) 153:711–22. 10.1053/j.gastro.2017.05.055 28596023

[37] Kuhnt K Fuhrmann C Köhler M Kiehntopf M Jahreis G. Dietary echium oil increases long-chain n-3 PUFAs, including docosapentaenoic acid, in blood fractions and alters biochemical markers for cardiovascular disease independently of age, sex, and metabolic syndrome. J Nutr. (2014) 144:447–60. 10.3945/jn.113.180802 24553695 PMC4083239

[38] Kucukgoncu S Zhou E Lucas KB Tek C. Alpha-lipoic acid (ALA) as a supplementation for weight loss: results from a meta-analysis of randomized controlled trials. Obesity Rev. (2017) 18:594–601. 10.1111/obr.12528 28295905 PMC5523816

[39] Forouhi NG Imamura F Sharp SJ Koulman A Schulze MB Zheng J. Association of plasma phospholipid n-3 and n-6 polyunsaturated fatty acids with type 2 diabetes: the EPIC-interact case-cohort study. PLoS Med. (2016) 13:e1002094. 10.1371/journal.pmed.1002094 27434045 PMC4951144

[40] Meyer BJ Groot RHM. Effects of omega-3 long chain polyunsaturated fatty acid supplementation on cardiovascular mortality: the importance of the dose of DHA. Nutrients. (2017) 9:1305. 10.3390/nu9121305 29189735 PMC5748755

[41] James MJ Ursin VM Cleland LG. Metabolism of stearidonic acid in human subjects: comparison with the metabolism of other n-3 fatty acids. Am J Clin Nutr. (2003) 77:1140–5. 10.1093/ajcn/77.5.1140 12716664

[42] Fukuda S Ohno H. Gut microbiome and metabolic diseases. Semin Immunopathol. (2014) 36:103–14. 10.1007/s00281-013-0399-z 24196453

[43] Jia X Xu W Zhang L Li X Wang R Wu S. Impact of gut microbiota and microbiota-related metabolites on hyperlipidemia. Front Cell Infect Microbiol. (2021) 11:634780. 10.3389/fcimb.2021.634780 34490132 PMC8417472

[44] Wei F Liu Y Bi C Chen S Wang Y Zhang B. Nostoc sphaeroids Kütz ameliorates hyperlipidemia and maintains the intestinal barrier and gut microbiota composition of high-fat diet mice. Food Science Nutr. (2020) 8:2348–59. 10.1002/fsn3.1521 32405392 PMC7215204

[45] Hills RD Pontefract BA Mishcon HR Black CA Sutton SC Theberge CR. Gut microbiome: profound implications for diet and disease. Nutrients. (2019) 11:1613. 10.3390/nu11071613 31315227 PMC6682904

[46] Zhu L Sha L Li K Wang Z Wang T Li Y. Dietary flaxseed oil rich in omega-3 suppresses severity of type 2 diabetes mellitus via anti-inflammation and modulating gut microbiota in rats. Lipids Health Dis. (2020) 19:20. 10.1186/s12944-019-1167-4 32028957 PMC7006389

[47] Wang F Zhu H Hu M Wang J Xia H Yang X. Perilla oil supplementation improves hypertriglyceridemia and gut dysbiosis in diabetic KKAy mice. Mol Nutr Food Res. (2018) 62:e1800299. 10.1002/mnfr.201800299 30358922 PMC6646911

[48] Kriss M Hazleton KZ Nusbacher NM Martin CG Lozupone CA. Low diversity gut microbiota dysbiosis: drivers, functional implications and recovery. Curr Opin Microbiol. (2018) 44:34–40. 10.1016/j.mib.2018.07.003 30036705 PMC6435260

[49] Boets E Gomand SV Deroover L Preston T Vermeulen K De Preter V. Systemic availability and metabolism of colonic-derived short-chain fatty acids in healthy subjects: a stable isotope study. J Physiol. (2017) 595:541–55. 10.1113/JP272613 27510655 PMC5233652

[50] Canfora EE Jocken JW Blaak EE. Short-chain fatty acids in control of body weight and insulin sensitivity. Nat Rev Endocrinol. (2015) 11:577–91. 10.1038/nrendo.2015.128 26260141

[51] Maji A Misra R Dhakan DB Gupta V Mahato NK Saxena R. Gut microbiome contributes to impairment of immunity in pulmonary tuberculosis patients by alteration of butyrate and propionate producers. Environ Microbiol. (2018) 20:402–19. 10.1111/1462-2920.14015 29322681

[52] Oluwagbemigun K O'Donovan AN Berding K Lyons K Alexy U Schmid M. Long-term dietary intake from infancy to late adolescence is associated with gut microbiota composition in young adulthood. Am J Clin Nutr. (2021) 113:647–56. 10.1093/ajcn/nqaa340 33471048 PMC7948843

[53] Liu F Li P Chen M Luo Y Prabhakar M Zheng H. Fructooligosaccharide (FOS) and galactooligosaccharide (GOS) increase bifidobacterium but reduce butyrate producing bacteria with adverse glycemic metabolism in healthy young population. Sci Rep. (2017) 7:11789. 10.1038/s41598-017-10722-2 28924143 PMC5603605

[54] Song Y Wu MS Tao G Lu MW Lin J Huang JQ. Feruloylated oligosaccharides and ferulic acid alter gut microbiome to alleviate diabetic syndrome. Food Res Int. (2020) 137:109410. 10.1016/j.foodres.2020.109410 33233097

[55] Kulecka M Fraczek B Mikula M Zeber-Lubecka N Karczmarski J Paziewska A. The composition and richness of the gut microbiota differentiate the top Polish endurance athletes from sedentary controls. Gut Microbes. (2020) 11:1374–1384. 10.1080/19490976.2020.1758009 32401138 PMC7524299

[56] Naderpoor N Mousa A Gomez-Arango LF Barrett HL Dekker Nitert M de Courten B. Faecal microbiota are related to insulin sensitivity and secretion in overweight or obese adults. J Clin Med. (2019) 8:452. 10.3390/jcm8040452 30987356 PMC6518043

[57] Citronberg JS Curtis KR White E Newcomb PA Newton K Atkinson C. Association of gut microbial communities with plasma lipopolysaccharide-binding protein (LBP) in premenopausal women. ISME J. (2018) 12:1631–41. 10.1038/s41396-018-0064-6 29434315 PMC6018759

[58] Liu Y Jiang H. Compositional differences of gut microbiome in matched hormone-sensitive and castration-resistant prostate cancer. Transl Androl Urol. (2020) 9:1937–44. 10.21037/tau-20-566 33209658 PMC7658119

[59] de Souza AZ Zambom AZ Abboud KY Reis SK Tannihão F Guadagnini D. Oral supplementation with L-glutamine alters gut microbiota of obese and overweight adults: a pilot study. Nutrition. (2015) 31:884–9. 10.1016/j.nut.2015.01.004 25933498

[60] Liu H Pan LL Lv S Yang Q Zhang H Chen W. Alterations of gut microbiota and blood lipidome in gestational diabetes mellitus with hyperlipidemia. Front Physiol. (2019) 10:1015. 10.3389/fphys.2019.01015 31447702 PMC6691352

[61] Wu H Esteve E Tremaroli V Khan MT Caesar R Mannerås-Holm L. Metformin alters the gut microbiome of individuals with treatment-naive type 2 diabetes, contributing to the therapeutic effects of the drug. Nat Med. (2017) 23:850–8. 10.1038/nm.4345 28530702

[62] Vieira-Silva S Falony G Belda E Nielsen T Aron-Wisnewsky J Chakaroun R. Statin therapy is associated with lower prevalence of gut microbiota dysbiosis. Nature. (2020) 581:310–5. 10.1038/s41586-020-2269-x 32433607

