# Supplementary material for: Effect of Plant-Derived n-3 Polyunsaturated Fatty Acids on Blood Lipids and Gut Microbiota: A Double-Blind Randomized Controlled Trial
Source: Front Nutr. 2022 Feb 11;9:830960. doi: 10.3389/fnut.2022.830960 (PMC8873928; doi:10.3389/fnut.2022.830960)
Supplement: Supplementary file 1 [file Data_Sheet_1.docx]

**Supplementary material**

Effect of plant-derived n-3 polyunsaturated fatty acids on blood lipids and gut microbiota: a double-blind randomized controlled trial

Hongjie Liu, Xiaoqin Li, Yalun Zhu, Yue Huang, Qin Zhang, Shan Lin, Can Fang, Linyan Li, Yanling Lv, Wenhua Mei, Xiaolin Peng, Jiawei Yin, Liegang Liu

**Supplementary Table 1** Content of oil capsule in control and intervention group.

**Supplementary Table 2** Changes in dietary intake of nutrients in subjects at baseline, 6 weeks and 12 weeks.

**Supplementary Table 3** Significantly changed predicted biochemical pathways during the intervention.

**Supplementary Figure 1** Clustering of gut microbiota into enterotypes at genus-level using Jensen-Shannon distance.

**Supplementary Figure 2** Changes of relative abundance of gut microbiota composition before and after diet intervention.

**Supplementary Table 1** Content of oil capsule in control and intervention group.

|  | **Fatty acid** | **Name** | **Intervention group^a^** | **Control group^b^** |
| --- | --- | --- | --- | --- |
| Saturated fatty acid (SFA) | C14:0 | myristic acid | 0.04 | 0.04 |
|  | C16:0 | palmitic acid | 5.89 | 10.70 |
|  | C18:0 | stearic acid | 3.21 | 1.70 |
|  | C20:0 | arachidic acid | 0.31 | 0.39 |
|  | C22:0 | behenic acid | 0.11 | 0.13 |
|  | C24:0 | lignoceric acid | 0.06 | 0.14 |
|  | Total SFA |  | 9.62 | 13.09 |
| Monounsaturated fatty acid (MUFA) | C18:1n9 | oleic acid | 12.80 | 28.50 |
|  | C20:1n9 | cetoleic acid | 2.21 | 0.27 |
|  | C22:1n9 | sinapic acid | 0.47 | 0.11 |
|  | C24:1n9 | nervonic acid | 0.22 | 0 |
|  | Total MUFA |  | 15.70 | 28.88 |
| n-6 Polyunsaturated fatty acid (PUFA) | C18:2n6 | linoleic acid | 17.60 | 44.40 |
|  | C18:3n6 | gamma linolenic acid | 7.26 | 0 |
|  | Total n-6 PUFA |  | 24.86 | 44.40 |
| n-3 Polyunsaturated fatty acid | C18:3n3 | alpha linolenic acid (ALA) | 29.70 | 0.56 |
|  | C18:4n3 | stearidonic acid (SDA) | 11.50 | 0 |
|  | Total n-3 PUFA |  | 41.20 | 0.56 |

^a^ Mixture of echium oil, camelina oil, and safflower seed oil. ^b^ Corn oil.

**Supplementary Table 2** Changes in dietary intake of nutrients in subjects at baseline, week 6 and week 12. ^a^

| Parameter | Changes within group ^a^ | | | | | | Between groups ^a^ | |
| --- | --- | --- | --- | --- | --- | --- | --- | --- |
|  | Control Group (n = 25) | | | Intervention Group (n = 26) | | | Changes | P value |
|  | Baseline | Changes in week 6 | Changes in week 12 | Baseline | Changes in week 6 | Changes in week 12 |  |  |
| Energy (kcal) | 2050.6 (1875.0, 2226.2) | -3.7 (-177.2, 169.9) | -5.2 (-165.4, 155.1) | 1977.8 (1805.6, 2150.0) | 48.5 (-111.8, 208.7) | 2.38 (-148.0, 152.8) | 7.55 (-212.2, 227.3) | 0.95 |
| Carbohydrate (g) | 294.37 (261.91, 326.82) | -11.93 (-40.46, 16.60) | -16.50 (-45.08, 12.08) | 287.56 (255.73, 319.38) | -11.23 (-37.60, 15.14) | -19.75 (-46.58, 7.08) | -3.25 (-42.45, 35.95) | 0.87 |
| Carbohydrate (E%) | 0.57 (0.54, 0.60) | 0.02 (-0.05, 0.01) | -0.03 (-0.06, 0.01) | 0.58 (0.55, 0.61) | -0.03 (-0.06, -0.01)* | -0.04 (-0.08, -0.01)* | 0.02 (-0.03, 0.07) | 0.51 |
| Protein (g) | 67.71 (60.22, 75.21) | -2.19 (-10.22, 5.84) | -1.04 (-9.23, 7.14) | 65.86 (58.51, 73.21) | -2.30 (-9.73, 5.13) | -2.23 (-9.91, 5.45) | -1.19 (-12.41, 10.04) | 0.83 |
| Protein (E%) | 0.13 (0.12, 0.14) | 0.00 (-0.01, 0.00) | 0.00 (-0.01, 0.01) | 0.13 (0.12, 0.14) | -0.01 (-0.02, 0.00) | 0.00 (-0.01, 0.01) | 0.00 (-0.02, 0.01) | 0.77 |
| Fat (g) | 68.73 (61.95, 75.51) | 5.07 (-3.54, 13.68) | 5.23 (-4.35, 14.81) | 65.50 (58.85, 72.14) | 10.73 (2.76, 18.70)** | 9.53 (0.53, 18.52)* | 4.29 (-8.85, 17.43) | 0.51 |
| Fat (E%) | 0.31 (0.28, 0.33) | 0.02 (-0.01, 0.05) | 0.02 (-0.01, 0.05) | 0.30 (0.28, 0.33) | 0.04 (0.01, 0.06)** | 0.04 (0.01, 0.07)* | 0.02 (-0.02, 0.07) | 0.29 |
| SFA (g/d) | 9.03 (7.65, 10.42) | -1.42 (-3.03, 0.20) | -0.51 (-1.91, 0.89) | 8.38 (7.02, 9.74) | -1.03 (-2.53, 0.47) | 0.64 (-0.68, 1.95) | 1.14 (-0.78, 3.07) | 0.24 |
| MUFA (g/d) | 17.39 (15.38, 19.39) | -2.19 (-4.26, -0.12) | -0.88 (-2.81, 1.05) | 16.13 (14.16, 18.10) | -1.31 (-3.24, 0.61) | 1.43 (-0.38, 3.24) | 2.31 (-0.33, 4.95) | 0.09 |
| PUFA (g/d) | 17.03 (15.68, 18.38) | 0.32 (-1.90, 2.54) | 0.35 (-1.45, 2.14) | 16.17 (14.84, 17.49) | 2.13 (0.06, 4.19)* | 3.39 (1.71, 5.07)* | 3.04 (0.59, 5,50) | 0.02 |
| Fiber (g) | 8.74 (5.78, 11.70) | -0.33 (-3.29, 2.63) | -1.01 (-4.45, 2.43) | 10.25 (7.35, 13.16) | -1.72 (-4.49, 1.04) | -1.26 (-4.49, 1.97) | -0.25 (-4.97, 4.47) | 0.92 |

Data are mean (lower confidence limit, upper confidence limit). Dietary intake of nutrients was obtained at week 0, representing baseline, and weeks 6 and 12 - baseline, representing change from baseline. Significant difference from baseline (*, P < 0.05; **, P < 0.01). ^a^ Mean, confidence limits, and P values determined using repeated-measures least squares means in PROC MIXED of SAS 9.4 with all available data. SFA, saturated fatty acid; MUFA, monounsaturated fatty acid; PUFA, polyunsaturated fatty acid.

**Supplementary Table 3** Significantly changed predicted biochemical pathways during the intervention.

| Pathway_ID | Level3 | Level2 | Level1 | Control group | | Intervention group | |
| --- | --- | --- | --- | --- | --- | --- | --- |
|  |  |  |  | *P* value | FDR p value | P value | FDR p value |
| ko00190 | Oxidative phosphorylation | Energy metabolism | Metabolism | 0.542 | 1.000 | 0.004 | 0.134 |
| ko00250 | Alanine, aspartate and glutamate metabolism | Amino acid metabolism | Metabolism | 0.200 | 0.987 | 0.001 | 0.124 |
| ko00261 | Monobactam biosynthesis | Biosynthesis of other secondary metabolites | Metabolism | 0.578 | 1.000 | 0.006 | 0.158 |
| ko00362 | Benzoate degradation | Xenobiotics biodegradation and metabolism | Metabolism | 1.000 | 1.000 | 0.004 | 0.134 |
| ko00450 | Selenocompound metabolism | Metabolism of other amino acids | Metabolism | 0.458 | 1.000 | 0.013 | 0.197 |
| ko00460 | Cyanoamino acid metabolism | Metabolism of other amino acids | Metabolism | 0.120 | 0.987 | 0.001 | 0.124 |
| ko00472 | D-Arginine and D-ornithine metabolism | Metabolism of other amino acids | Metabolism | 0.113 | 0.987 | 0.014 | 0.197 |
| ko00510 | N-Glycan biosynthesis | Glycan biosynthesis and metabolism | Metabolism | 0.711 | 1.000 | 0.011 | 0.197 |
| ko00633 | Nitrotoluene degradation | Xenobiotics biodegradation and metabolism | Metabolism | 0.353 | 1.000 | 0.012 | 0.197 |
| ko00908 | Zeatin biosynthesis | Metabolism of terpenoids and polyketides | Metabolism | 0.895 | 1.000 | 0.002 | 0.124 |
| ko00940 | Phenylpropanoid biosynthesis | Biosynthesis of other secondary metabolites | Metabolism | 0.979 | 1.000 | 0.001 | 0.124 |
| ko00983 | Drug metabolism - other enzymes | Xenobiotics biodegradation and metabolism | Metabolism | 0.090 | 0.987 | 0.002 | 0.124 |
| ko01524 | Platinum drug resistance | Drug resistance: Antineoplastic | Human Diseases | 0.148 | 0.987 | 0.002 | 0.124 |
| ko02010 | ABC transporters | Membrane transport | Environmental Information Processing | 0.508 | 1.000 | 0.014 | 0.197 |
| ko02060 | Phosphotransferase system (PTS) | Membrane transport | Environmental Information Processing | 0.596 | 1.000 | 0.014 | 0.197 |
| ko03020 | RNA polymerase | Transcription | Genetic Information Processing | 0.474 | 1.000 | 0.007 | 0.161 |
| ko03070 | Bacterial secretion system | Membrane transport | Environmental Information Processing | 0.080 | 0.987 | 0.004 | 0.134 |
| ko04122 | Sulfur relay system | Folding, sorting and degradation | Genetic Information Processing | 0.220 | 0.987 | 0.009 | 0.193 |
| ko04146 | Peroxisome | Transport and catabolism | Cellular Processes | 0.853 | 1.000 | 0.005 | 0.158 |
| ko04210 | Apoptosis | Cell growth and death | Cellular Processes | 0.474 | 1.000 | 0.007 | 0.159 |
| ko04216 | Ferroptosis | Cell growth and death | Cellular Processes | 0.874 | 1.000 | 0.012 | 0.197 |
| ko04974 | Protein digestion and absorption | Digestive system | Organismal Systems | 0.874 | 1.000 | 0.006 | 0.158 |

**
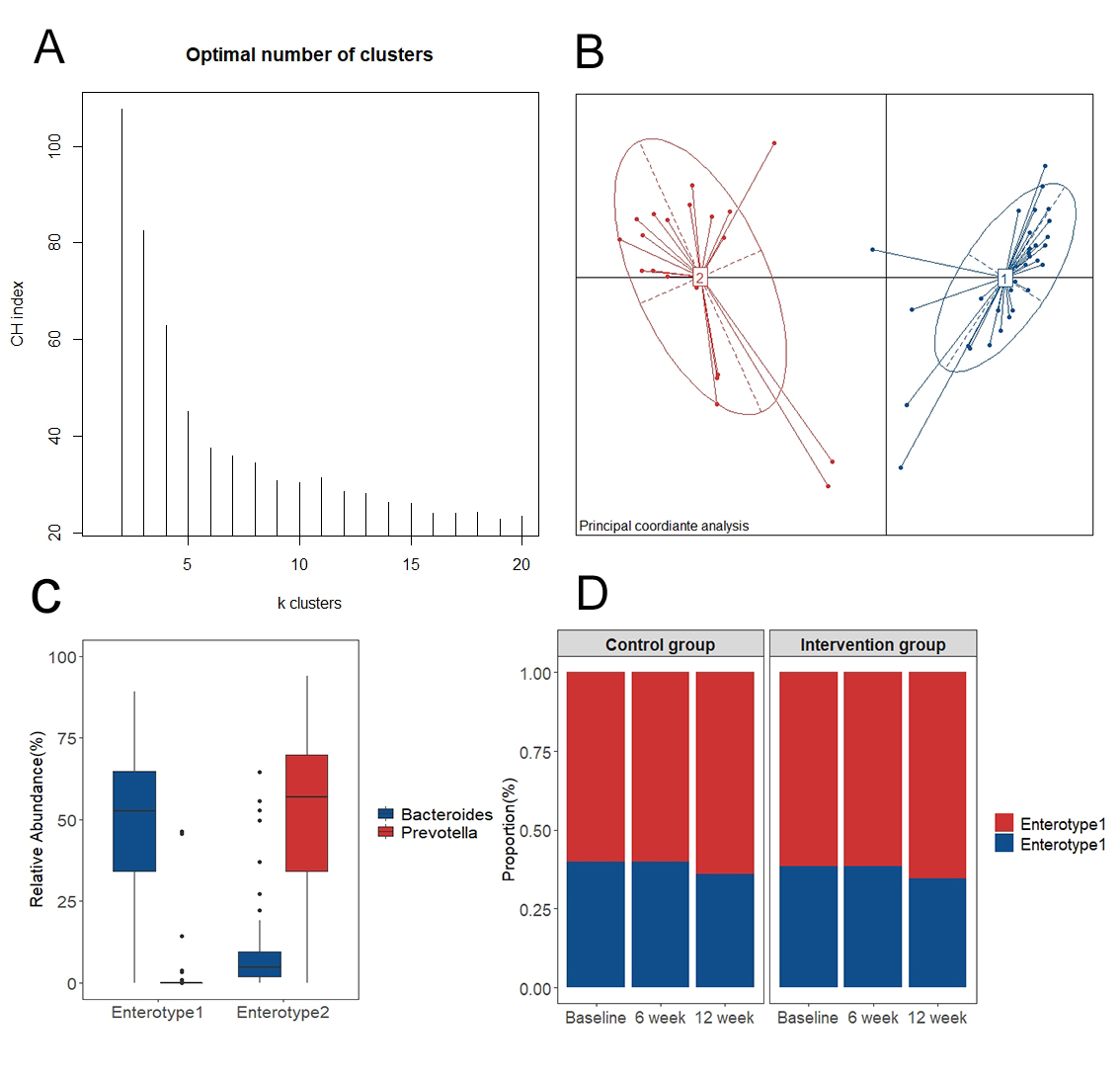
Supplementary Figure 1 Clustering of gut microbiota into enterotypes at genus-level using Jensen-Shannon distance.**

(A) Two clusters exist most naturally in the dataset by the PAM method. The x-axis shows the cluster number, the y-axis shows the Calinski-Harabasz (CH) index. (B) Clustering of the first two principal components. (C) Relative abundance of bacterial taxa characteristic of each enterotype. Boxes represent the IQR and the line within represents the median. whiskers represent 10-90 percentiles, dot represents outliers that are past the ends of the whiskers. (D) Proportions of enterotypes in each group before and after the intervention. No statistically significant changes were seen in each group before and after the intervention.

**
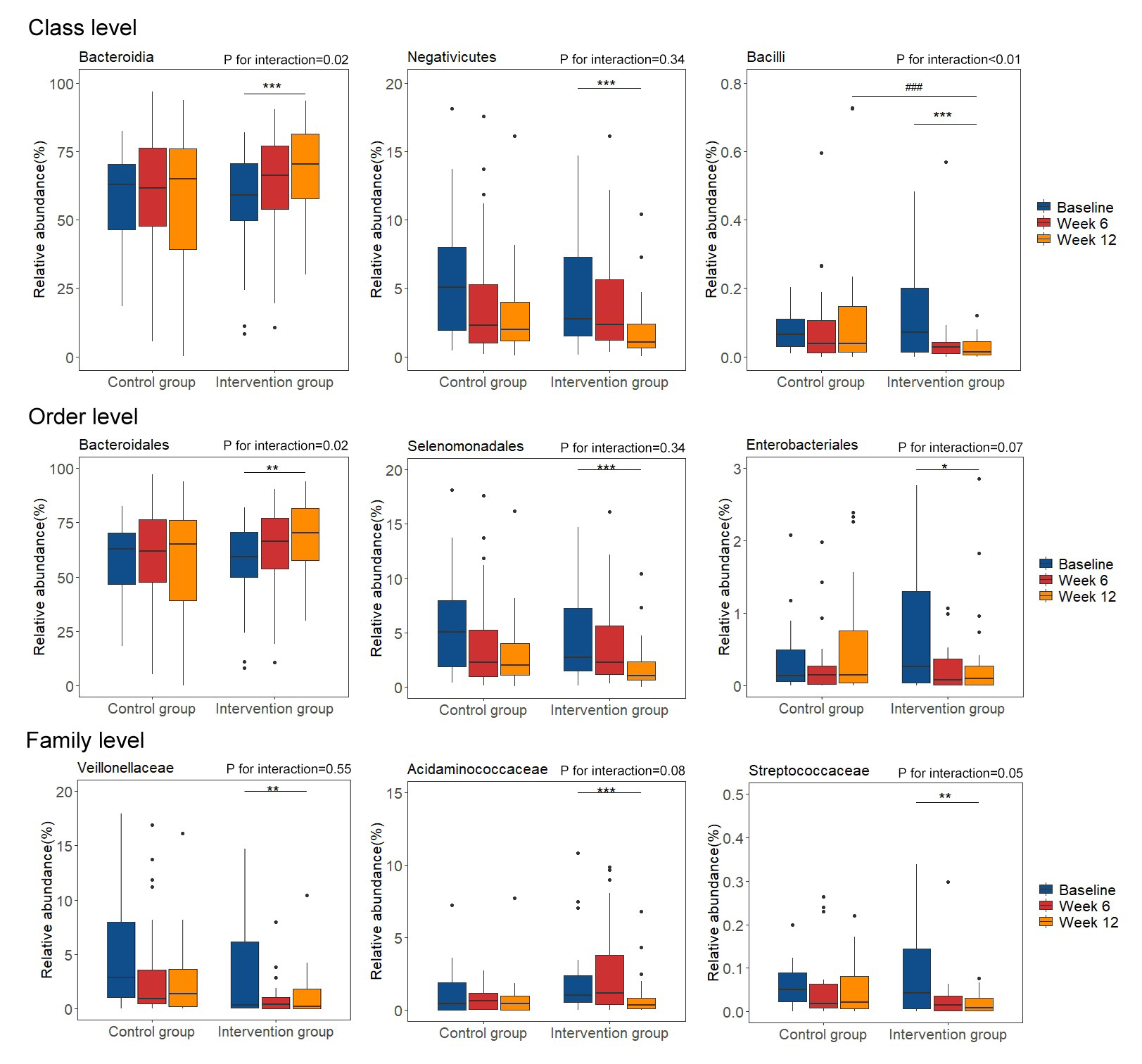
Supplementary Figure 2. Changes of relative abundance of gut microbiota composition before and after diet intervention.**

* P <0.05, ** P <0.01, *** P <0.001 vs baseline of the same group. ^#^ P <0.05, ^##^ P <0.01, ^###^ P <0.001 vs control group at the same time point.
